# Mapping evidence of socio-cultural factors in intimate partner violence among young women: a scoping review protocol

**DOI:** 10.1186/s13643-019-1234-y

**Published:** 2019-12-06

**Authors:** Maria Suzana B. Maguele, Nelisiwe Khuzwayo

**Affiliations:** 1 Instituto Superior de Ciências de Saúde - ISCISA (High Institute for Health Sciences), Maputo, Mozambique; 2 Discipline of Public Health Medicine, School of Nursing and Public Health, College of Health Sciences, University of KwaZulu-Natal, Durban, South Africa

**Keywords:** Intimate partner violence, Violence against women, Socio-cultural factors, Dating violence, Domestic violence

## Abstract

**Background:**

Intimate partner violence among young women continues to be a worldwide concern. Globally, a considerable number of studies reported numerous factors that influence intimate partner violence among the young. The proposed scoping review aims to map available evidence of socio-cultural factors influencing intimate partner violence among young women.

**Methods:**

We will conduct a scoping review to explore, describe and map literature on socio-cultural factors influencing intimate partner violence among young women. The search strategy for this scoping review study will involve electronic databases including PubMed, Web of Knowledge, Science Direct, EBSCOHost (PubMed, CINAHL with Full Text, MEDLINE), Google Scholar, BioMed Central and World Health Organization library. Articles will also be searched through the “Cited by” search as well as citations included in the reference lists of included articles. Keyword searches will be used, and two independent reviewers will be screening titles, abstracts and full articles; where there are disputes between the two reviewers, a third reviewer will intervene. Thematic analysis will be employed to present the narrative account of the review.

**Discussion:**

Understanding socio-cultural factors influencing intimate partner violence among young women is critical. This will enable researchers to map existing literature, map research gaps and guide future research.

**Systematic review registration:**

PROSPERO (CRD42018116463)

## Background

Intimate partner violence (IPV) as defined by the World Health Organization (WHO) is a global phenomenon of violent acts that include physical, sexual and psychological/emotional abuse by a current or former intimate partner [1]. The WHO estimates that one in three (>30%) women experience violence from their partners globally, and the region of Sub-Saharan Africa (SSA) has shown higher prevalence where the statistics point to 36.6% [2, 3].

IPV among young women have been the subject of intense debate within the scientific community. Worldwide, IPV among ever-partnered young women aged 20–24 years was estimated to be around 31.6% and 29.4% among women aged 15–24 years in 2013 [4]. Of concern is the early exposure of young women to IPV since they are in the transitional stages of development [3, 5].

There are numerous factors influencing IPV among young women including HIV-positive status, level of education, economic status [6], alcohol abuse and socio-cultural factors [7]. There is a consensus among social scientists that young women who are subjected to IPV are more likely to acquire negative health outcomes including unwanted pregnancy, abortion, sexually transmitted infections including HIV, injuries or being murdered [8]. While factors influencing IPV among women are well documented, it is particularly important to understand socio-cultural factors influencing IPV among young women. Knowing that this is potencially the group of population with other risk factors including the risk of IPV, therefore the impact of IPV could be relatevely higher [5, 9]. Socio-cultural factors are beliefs, customs and practices within cultures and societies that affect the thoughts, feelings and behaviours of the community members [10]. Although socio-cultural factors influencing IPV are evident among older women, there are fewer studies focusing on younger women [7].

This information will be vital for researchers, governments and non-governmental organizations for the development of context-based primary interventions and policies. Further, the information generated from this review will benefit health authorities, health care workers, academics and general public. It will also be useful for educational purposes.

Therefore, this scoping review seeks to:

❖ Map existing types of socio-cultural factors influencing IPV among young women in Sub-Saharan Africa

❖ Map the extent in which socio-cultural factors promote IPV victimization of young women

❖ Determine the nature and quality of studies reporting evidence of socio-cultural factors on the intimate partner violence among young women

The findings from this review will enable the researchers to examine the extent, range and nature of research activity on IPV and socio-cultural factors among young women in Sub-Saharan Africa. The findings will also enable the researchers to identify and describe the context in which young women experience IPV as well as the main reasons for it.

## Methodology

The current scoping review protocol is registered and published in PROSPERO, an international prospective register for systematic reviews under the following registration number: CRD42018116463.

The framework adopted for conducting the proposed review is by Arksey and O’Malley [11]. Briefly, the framework involves (i) identifying the research question, (ii) identifying relevant studies, (iii) study selection, (iv) charting the data and (v) collating, summarizing and reporting the results. This scoping review will include quality appraisal of studies.

### Identifying the research question

What is the available evidence of socio-cultural factors in IPV among young women? The research sub-questions are:

1. Is there evidence of types of socio-cultural practices on IPV among young women in Sub-Saharan Africa?
2. Is there evidence that shows that socio-cultural factors contribute to IPV victimization among young women in Sub-Saharan Africa?
3. Is there evidence of the nature and quality of the studies reporting evidence of socio-cultural factors on IPV among young women in Sub-Saharan Africa?

#### Eligibility Criteria

The study will use an amended PICOS (Population, Intervention, Comparison, Outcomes and Study setting) framework to determine the eligibility of the research questions (Table 1).

**Table 1:** PICOS framework

Criteria	Determinants
Population	Young women aged 15–24 experiencing IPV
Intervention	Socio-cultural factors, intimate partner violence
Comparison	N/A
Outcomes	Intimate partner violence, socio-cultural factors
Study setting	Sub-Saharan Africa

#### Inclusion criteria

We will include

- Studies that show evidence on socio-cultural factors on IPV
- Studies that show evidence of intervention on IPV
- Studies that include the following outcomes: IPV, socio-cultural factors, morbidity/mortality and health effects of IPV
- Studies done in SSA

#### Exclusion criteria

We will exclude

- Studies reporting evidence of IPV among women under the age of 15 and women above the age of 24
- Studies that were published before 2008
- Studies on non-partner intimate violence
- Studies evidencing intervention on non-male intimate partner violence against women

### Identifying relevant studies

Primary research articles published in peer-reviewed journals, review articles and grey literature that addresses the main research question will be included in this study. All study designs will be included in this review. Databases that the study will use to source literature include PubMed, CINAHL with Full Text, Health Sources MEDLINE, World Health Organization (WHO) and governmental websites which would be searched for policies and reports. This study will also use reference lists and existing networks such as organizations and conferences to source relevant literature. The search terms will include “Intimate partner violence”, “Factors influencing intimate partner violence”, “socio-cultural factors on the intimate partner violence”, “dating violence”, and “domestic violence”.

This review will search studies that were published in any language; by not restricting languages of publications, all languages will thus be included.

The search strategy will be first piloted to determine the validity and reliability of the criteria of the study selection (Table 2). The pilot search results are presented in Appendix 1 (Table 4). The engine used is the MEDLINE database via EBSCOHost using the MeSH terms for searching.

**Table 2:** Database search record

Date of search - Search engine - Keyword search - Number of articles found - Number of articles eligible	

### Study selection

**Fig. 1:**
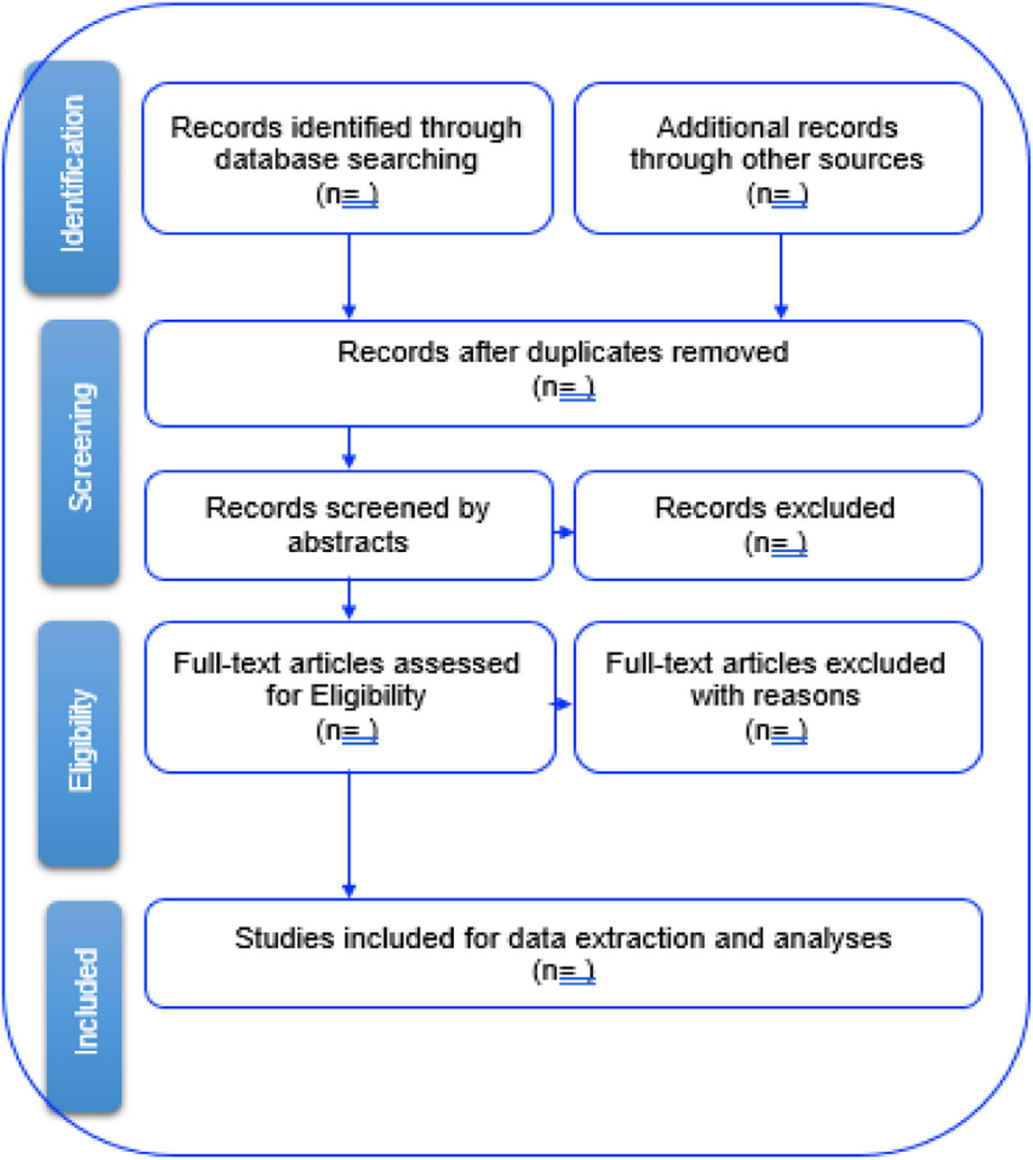
Study selection procedure

The study will conduct a comprehensive title screening by searching and uploading all literature search results on Endnote X7 software (Fig. 1). All the studies that do not address the research questions will be excluded together with all the duplicates. The reviewers will seek assistance from the UKZN library services for articles that are difficult to find. The reviewers will also contact authors to request full copies of the included articles that are not available via the databases and UKZN library. The final Endnote database will be shared among the review team for abstract screening; at this stage, two independent reviewers will screen the abstracts and full articles, with guidance from the inclusion criteria. Copies of full articles for eligible studies will be obtained and maintained for data extraction.

### Charting the data

The researchers will use a standardized data extraction sheet (Appendix 2, Table 5). The sheet will include bibliographic details, study design, number of participants, intervention, study setting, significant findings and conclusions for the primary and the secondary outcomes of the intervention (Table 3).

**Table 3:** Data charting form

Author and date	
Study design	
Study setting	
Population	
Overage age	
Sample size	
Aims	
Interventions	
Outcomes	
Key findings	
Conclusion	
Comments	

### Collating, summarizing and reporting the results

The researchers will present a narrative account of the findings from the existing literature through a thematic content analysis of the extracted literature. The themes will be structured around the following interned outcomes which will be coded by all authors independently: socio-cultural, economic and behavioural factors, and experiences and reasons for acceptance of IPV. The emerging theme will also be reported. In aiding the thematic analysis, NVivo version 10 shall be used. The subsequent processes will be followed:

Coding

Categorize codes into major themes

Build theme-related themes (cut-and-paste technique)

Display data

Identify patterns in the data and identify sub-themes

Summarize

### Quality assessment

Authors will interrogate the resulting themes and critically examine their relationship to the research questions. Authors will also scrutinize the meanings of the findings as they relate to the overall aim of the study and the implications for future research. A quality appraisal tool which focuses on the study methods, the Mixed Method Quality Appraisal Tool (MMAT), version 2011, will be used [12]. The tool will be used to examine the quality of an article looking at the following aspects: the appropriateness of the aim of the study, adequacy and methodology, study design, participant recruitment, data collection, data analysis, presentation of findings, authors’ discussions and conclusions.

## Discussion

The scoping review will be conducted as a first part of the study focusing on socio-cultural factors influencing IPVAYW in Mozambique. This scoping review aims to map evidence of socio-cultural factors in IPV among young women in SSA. The findings of this review will identify the extent to which socio-cultural factors among young women influence IPV. The purpose is to establish the extent of existing research on socio-cultural factors on IPV in SSA. Although studies on factors of IPV are taking place in these countries [7], there is still a scarcity of evidence on types of socio-cultural factors on the IPVAYW [1, 10]. The researchers will limit the research to include published studies from 2008 to 2019. A 10-year literature search is more likely to yield a comprehensive and balanced account of previous and current research in the area and to capture past as well as emerging perspectives on interventions on socio-cultural factors on IPV. This review will exclude studies that report evidence on non-partner intimate violence, as the focus is on intimate partner violence. The researchers therefore anticipate finding relevant literature on IPV in SSA. The results will provide documented evidence on socio-cultural factors on the IPVAYW and will help identify requirement priorities for primary research in this area. Due to how this study proposes to guide future research, the dissemination plans include presentations on public health institutions, local stakeholders, conference presentations and publication in journals. The review will also identify priorities for primary research and future research.

## Data Availability

All data generated or analysed during this study will be included in the published systematic review article.

## Appendix 1

**Table 4:** Results of the pilot search

Date of search	Search engine used	Keyword searched	Number of publications retrieved
8 May 2019	MEDLINE via EBSCOHost	(“intimate partner violence”) AND (“sociocultural factors”) AND (“dating violence”) AND (“violence against Human”) AND (“domestic violence”) AND (“Young women”) AND (“Sub Saharan Africa”) Published date: 2008 Jan 1 to 2019 Dec 31 Source types: academic journals	7570

## Appendix 2

**Table 5:** Sample data extraction form

Study details	
Author/year	
Objectives	
Participants (characteristics/total number)	
Setting/context	
Interventions	
Search details	
Sources searched	
Years of included studies	
Number of studies included/types or studies included	
Country of origin of included studies	
Appraisal	
Appraisal instruments used	
Appraisal rating	
Analysis	
Method of analysis	
Outcome assessed	
Result findings	
Significance/direction	

## Abbreviations

IPVAYW: Intimate partner violence among young women
SSA: Sub-Saharan Africa
WHO: World Health Organization
YW: Young woman

## References

[1] Organization World Health. Responding to intimate partner violence and sexual violence against women. WHO clinical and policy guidelines. World Health Organization; 2013. 24354041

[2] Women U, UNICEF. International technical guidance on sexuality education: an evidence-informed approach: UNESCO Publishing; 2018.

[3] García-Moreno C, Pallitto C, Devries K, Stöckl H, Watts C, Abrahams N. Global and regional estimates of violence against women: prevalence and health effects of intimate partner violence and non-partner sexual violence: World Health Organization; 2013.

[4] Stöckl H, March L, Pallitto C, Garcia-Moreno C (2014). Intimate partner violence among adolescents and young women: prevalence and associated factors in nine countries: a cross-sectional study. BMC Public Health.

[5] Viner RM, Ozer EM, Denny S, Marmot M, Resnick M, Fatusi A (2012). Adolescence and the social determinants of health. Lancet.

[6] Jewkes R (2002). Intimate partner violence: causes and prevention. Lancet.

[7] Jewkes R, Levin J, Penn-Kekana L (2002). Risk factors for domestic violence: findings from a South African cross-sectional study. Soc Sci Med.

[8] Campbell JC (2002). Health consequences of intimate partner violence. Lancet.

[9] Heise LL (1998). Violence against women: an integrated, ecological framework. Violence Against Women..

[10] Organization World Health (2009). Changing cultural and social norms that support violence.

[11] Arksey H, O’Malley L (2005). Scoping studies: towards a methodological framework. Int J Soc Res Methodol.

[12] Pluye P, Robert E, Cargo M, Bartlett G, O’cathain A, Griffiths F, et al. Proposal: a mixed methods appraisal tool for systematic mixed studies reviews. Montréal: McGill University. 2011;2:1-8.

